# Factors That May Impact the Noninvasive Measurement of Central Blood Pressure Compared to Invasive Measurement: The MATCHY Study

**DOI:** 10.3390/jpm12091482

**Published:** 2022-09-10

**Authors:** Chen Chi, Yi Lu, Yiwu Zhou, Jiaxin Li, Yawei Xu, Yi Zhang

**Affiliations:** 1 Department of Cardiology, Tongji University School of Medicine, Shanghai Tenth People’s Hospital, Shanghai 200072, China; 2 Department of Cardiology, The Sixth People’s Hospital of Nantong, Nantong 226001, China; 3 The Research Institute of Clinical Epidemiology, Tongji University School of Medicine, Shanghai 200072, China

**Keywords:** blood pressure measurement, central blood pressure, invasive blood pressure, calibration methods

## Abstract

Calibration affects central blood pressure (BP) estimation accuracy. Factors influencing the accuracy of noninvasive central BP measurement, type of calibration method implemented (systolic/diastolic BP or mean/diastolic BP), and type of BP measurement device used (devices using the transfer function method, directly measurement from the carotid artery, and the transfer function-like method), were investigated. Fifty participants (aged 62.4 ± 8.9 years) without overt heart diseases were recruited. Invasive aortic and radial BP was measured. Simultaneously, noninvasive central BP was measured using three types of devices. The mean invasive aortic BP was 127 ± 19/95 ± 14 mmHg. Noninvasive central BP tended to be slightly lower than invasive BP, though without statistical significance. The type of calibration method did not significantly influence the noninvasive cSBP measurements (*p* ≥ 0.24). Results from cuff-based devices were significantly lower than invasive measurements (*p* = 0.04). Multiple regression analyses showed that gender was significantly correlated with the accuracy of noninvasive cSBP measurement. In conclusion, noninvasive cSBP measurements are comparable to invasive measurements but might underestimate true cSBP. The type of device may affect the accuracy of measurement. Either of the two calibration methods is acceptable.

## 1. Introduction

Hypertension is one of the most challenging public health concerns worldwide. Current blood pressure (BP) management strategies, including BP measurement, cardiovascular risk assessment, BP targets, and treatment of hypertension, are based on brachial blood pressure [1]. It is undeniable that brachial BP is of great importance in clinical practice. However, from the physiological point of view, there is a significant discrepancy between brachial and central BP, and brachial values cannot represent the true pressure that target organs such as the heart, aorta, and kidney encounter. Many studies have shown that central BP, compared with brachial BP, is more closely associated with hypertension-mediated organ damage in populations with diverse characteristics [2,3,4], and better predicts cardiovascular events [5]. Though central BP is gaining more attention, the incremental prognostic value of central over brachial BP is not widely recognized, which might be owing to the inaccurate estimation of noninvasively measured central BP. A meta-analysis, which included 22 studies, showed that the mean difference between noninvasive and invasive measurement of central systolic BP (cSBP) was about −8 mmHg (95% confidence interval (CI): [−28.4, 12]) [6]. Though the absolute difference was not statistically significant, it was still too large to be ignored. Thus, improving the measurement accuracy of noninvasive central BP measurement is of utmost importance.

Several factors may influence the accuracy of noninvasive central BP measurement, for example, devices with different techniques and different calibration methods (such as the transfer function method using calibrated systolic BP (SBP)/diastolic BP (DBP) or the pressure equivalence method using mean calibrated BP (MBP)/DBP). Many noninvasive central BP estimation devices are available on the market, and a majority of them estimate central BP with the brachial cuff. There are currently three main strategies for noninvasive central BP measurement [7]: (1) measuring the radial waveform and estimating aortic BP with a transfer function (or the second systolic peak), which is represented by the SphygmoCor device; (2) measuring the carotid waveform and estimating the carotid BP, which is used by PulsePen device; (3) measuring the brachial waveform and estimating central BP with a transfer function-like method, represented by the brachial cuff-based Mobil-O-Graph device. All these techniques include two essential steps: waveform measurement and calibration. If the waveform is correctly recorded, characteristics of the type of calibration may have a great impact on the accuracy of noninvasive central BP measurement. A study focusing on calibration methods and BP measurement devices is necessary.

Therefore, we conducted a comparison study to investigate the potential impact of the factors mentioned above. Our study is divided into two parts. First, invasive and noninvasive central BP measurements were performed simultaneously using the standard protocol of each device. Second, noninvasively measured central BP was then calibrated using different calibration methods. The results were compared to the invasive central BP measurement to investigate the potential impact of calibration methods and BP measurement devices on the accuracy of noninvasive central BP measurements.

## 2. Materials and Methods

### 2.1. Study Design and Participants

**Figure 1:**
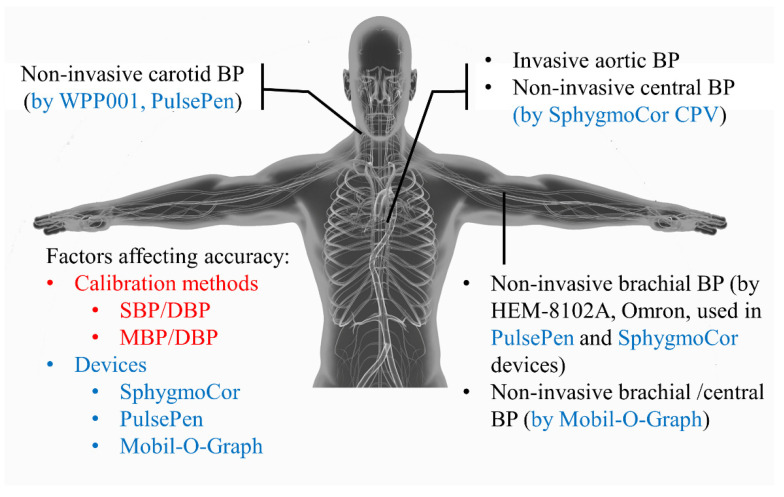
Study design. Factors (calibration methods and devices) that may impact the noninvasive measurement accuracy of central blood pressure are shown in red and blue, respectively. BP: blood pressure. SBP: systolic BP. DBP: diastolic BP. MBP: mean BP.

This study, named “coMparison between invasive and noninvasive assessment of blood pressure and cardiac function in HealthY participants: the MATCHY study,” was a registered single-center, cross-sectional study (registered number, NCT03372616). The present analysis aimed to investigate the potential impact of calibration methods and BP measurement devices on the accuracy of noninvasive central BP measurements (summarized in Figure 1). Fifty consecutive inpatients were recruited from the Department of Cardiology of Shanghai Tenth People’s Hospital for clinical characteristics collection, anthropometric measurement, and blood pressure measurement. The inclusion criteria were (1) ≥18 years old, (2) without the change of medication that may influence hemodynamics for 1 month, and (3) intending to undergo coronary angiography and left ventriculography. The exclusion criteria were (1) without sinus rhythm or with frequent premature beats, (2) diagnosed coronary artery disease, (3) diagnosed pulmonary vascular or parenchymal disease, (4) primary or secondary cardiomyopathy, (5) pericarditis, (6) moderate or severe valvular stenosis or insufficiency, (7) congenital heart disease, (8) heart transplantation, and (9) poor image quality of echocardiography. Noninvasive central BP measurement values using different calibration methods and devices were directly compared to the invasively measured values. All participants signed informed consent. This study was approved by the ethics committee of Shanghai Tenth People’s Hospital.

### 2.2. Anthropometric Measurements, Clinical Characteristics, and Laboratory Examinations

Body height and weight were measured by 1 nurse, and the body mass index was defined as the ratio of body weight (in kilograms) and squared body height (in meters). Clinical characteristics such as past medical history, family history of diseases, smoking and exercise habits, and pharmacological treatment were obtained with a standard questionnaire and confirmed with patients’ documents. Smoking was defined as a previous and/or current smoker. Laboratory examinations, including blood creatinine, troponin, fasting blood glucose, glycosylated hemoglobin, urinary albumin creatinine ratio, etc., were measured by the clinical laboratory in Shanghai Tenth People’s Hospital with a standard protocol. Hypertension was defined as noninvasive brachial measurements with SBP > 140 and/or DBP > 90 mmHg when obtained at least 2 times on 2 different days in the clinical setting, or if the participant was prescribed any anti-hypertensive agent. Diabetes was defined as fasting blood glucose >7.0 mmol or if the participant was prescribed any anti-diabetic drugs.

### 2.3. Invasive Blood Pressure Measurement

Before the invasive measurement of blood pressure, coronary angiography together with left ventriculography was performed to exclude patients with coronary artery disease or abnormal ejection fraction (lower than 50%). A 5F micromanometer tip catheter (Cordis, U.S.) was connected to the multipurpose polygraph (St. Jude Medical, U.S.), then calibrated with saline to be adjusted to the baseline. This catheter was inserted into the ascending aorta (2–3 cm from the aortic valve) from a 6F radial arterial sheath. Steady waveforms were recorded for at least 10 cardiac cycles. Subsequently, the catheter was retracted to the left common carotid artery to record carotid waveforms. Finally, the 6F radial arterial sheath was directly connected to the multipurpose polygraph with a pressure transducer, after the withdrawal of the catheter, to record the radial waveforms. Similar to the former steps, the sheath was calibrated and adjusted to the baseline, and then steady waveforms were recorded for at least 10 cardiac cycles. The average blood pressure of each site was calculated. The invasive aortic BP was used as the reference when evaluating the accuracy of noninvasive central BP measurement, and the invasive radial BP was used in calibration to test the influence of the BP site.

### 2.4. Noninvasive Blood Pressure Measurement

Noninvasive brachial blood pressure measurement (HEM-8102A, Omron, Japan) was performed 2 times simultaneously with invasive measurement in the supine position, and the average was used for further analysis. All noninvasive central BP measurements were performed simultaneously with invasive measurement or within 10 min after invasive measurement using a standard protocol in the cath lab in the supine position. Three devices were used for every participant—SphygmoCor (CPV with SphygmoCor SCOR-Px, SCOR-Vx, and SCOR-Mx embedded, AtCor Medical, Sydney, Australia), PulsePen (WPP001-ETT, DiaTecne, Milano, Italy), and Mobil-O-Graph (IEM, Aachen, Germany). These 3 devices were used in random order to avoid potential bias because of the time intervals between invasive and noninvasive measurements. Briefly, the SphygmoCor estimated central BP based on the transfer function by measuring radial waveforms with transcutaneous applanation tonometry and using calibrated noninvasive brachial SBP/DBP records (measured by the Omron device in our study). The PulsePen was similar to the SphygmoCor device but was only able to estimate central BP with carotid waveforms. The Mobil-O-Graph was a brachial cuff-based device with the transfer function-like method, and, with this device, the calibrated noninvasive brachial SBP/DBP was recorded by itself.

### 2.5. Calibration Methods of Noninvasive Aortic Blood Pressure Measurement

Noninvasive central SBP measurement, recorded by different devices, was further calibrated using various methods. For the SphygmoCor device, brachial BP was recorded and two calibration methods, SBP/DBP and MBP/DBP were used, respectively. For the PulsePen device, only one calibration method, the SBP/DBP method, was available in its software. For the Mobil-O-Graph device, our software (version 5.0, IEM, Germany) provided an estimated central BP automatically and did not provide access to the modification, only one calibration method (SBP/DBP method) was used as well.

### 2.6. Statistical Analysis

Continuous variables were expressed as mean ± SD when normally distributed, or as medium (quantiles) if not. Categorical variables were expressed as numbers (percentages). The paired t-test with Bonferroni adjustment and Bland–Altman method were used to compare invasive SBP measurement to each corresponding noninvasive SBP measurement determined by the standard protocol. For the comparison between invasive and noninvasive central SBP measurement using different calibration methods, 2 statistical methods were used. One method included directly comparing the absolute SBP value using analysis of variance (ANOVA) of repeated measures with Bonferroni adjustment. The second method included comparing paired differences of invasive and noninvasive central SBP measurement using one-way ANOVA with Bonferroni adjustment. Spearman correlation analyses were performed to test the association between invasive and each noninvasive central SBP measurement, and the Z-test was performed to compare these correlation coefficients. Then, all paired differences were aggregated and classified based on the potential influencing factors of our study, respectively, and compared to the reference “zero” using one-way ANOVA. Finally, full-model linear logistic regression was performed to assess the influence of age, gender, peripheral site, calibration method, and device on the noninvasive central BP measurement. All statistical analyses were performed with statistical software SAS version 9.3 (SAS Institute, Cary, NC, USA).

## 3. Results

### 3.1. Characteristics of Participants

A total of 50 participants were recruited in the present study, in which 23 participants were male, 11 participants were current smokers, and 7 participants were current drinkers. The mean age was 62.4 ± 8.9 years and the mean body mass index (BMI) was 25.1 ± 3.0 kg/m^2^. Table 1 shows the general characteristics of all participants.

### 3.2. Invasive and Noninvasive BP Measurement Parameters

**Figure 2:**
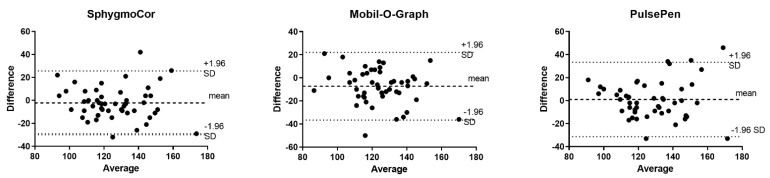
Bland-Altman plots. Bland-Altman plots were used to compare the invasive measure-ment and each corresponding noninvasive SBP measurement determined by standard protocol, namely the brachial systolic/diastolic calibration method.

Table 2 presents the mean invasive and noninvasive BP measurement parameters of all participants. Among the invasive measurements, aortic SBP was comparable to carotid SBP (*p* = 0.35), but it was significantly lower than brachial BP (*p* < 0.001). The invasive radial SBP measurement was extremely proximal to the noninvasive brachial BP measurement (*p* = 0.98). Among the noninvasive measurements, the aortic SBP recorded by SphygmoCor was similar to the SphygmoCor-measured carotid SBP (*p* = 0.10) but was slightly lower than the PulsePen-recorded carotid SBP (*p* = 0.002) and significantly higher than the Mobil-O-Graph-recorded aortic SBP (*p* = 0.006). However, the noninvasive brachial SBP measurement obtained by the Mobil-O-Graph device was significantly lower than the Omron device (126 ± 19 vs. 140 ± 19 mmHg, *p* < 0.001). A Bland–Altman plot was used to compare the invasive measurement and each corresponding noninvasive SBP measurement determined by standard protocol (Figure 2). Proportional bias was not observed using these three devices.

### 3.3. Central SBP Values Using Different Calibration Methods and Comparison with Invasive Measurements

**Figure 3:**
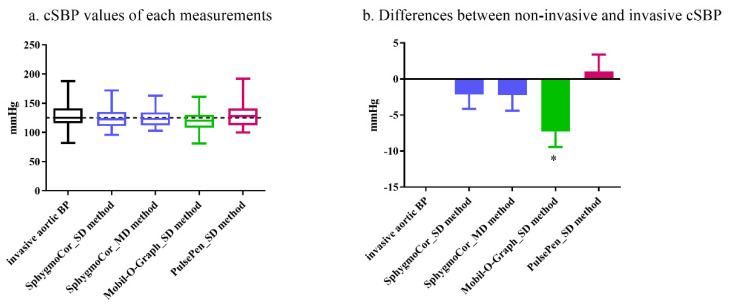
Central SBP values using different devices and calibration methods, and comparison with invasive measurements. Devices are shown in blue (SphygmoCor), green (Mobil-O-Graph), and red (PulsePen), separately. (**a**) the medium value, quartiles, maxima, and minima of inva-sive and noninvasive central SBP values using different devices and calibration methods. The dashed line indicates the medium of invasive aortic SBP value. (**b**) the difference between each noninvasive central SBP measurement and the invasive aortic SBP measurement (the invasive measurement was taken as reference “zero”). ANOVA with Bonferroni post hoc analysis was used to compare each noninvasive central SBP with the invasive aortic SBP. cSBP: central systolic blood pressure. SD: calibrated with the systolic/diastolic method. MD: calibrated with the mean/diastolic method. In our study, Mobil-O-Graph and PulsePen can only be calibrated with the systolic/dias-tolic blood pressure, thus it has only one group. *: *p* < 0.05.

Noninvasive central SBP measurements recorded by different devices were further calibrated using different methods, and these SBP values were then compared to the invasively measured values. The medium value, quartiles, maxima, and minima are shown in Figure 3a. ANOVA with Bonferroni post hoc analysis was used to compare each noninvasive central SBP measurement with the invasive aortic SBP measurement, with no significant difference noted in the results of this test (*p* ≥ 0.18). Next, the difference between each noninvasive central SBP measurement and the invasive aortic SBP measurement was calculated, and, subsequently, the differences were compared to the reference, zero. These differences are demonstrated in Figure 3b. The noninvasive central SBP measurement obtained by the SphygmoCor device, independent of the type of calibration method used, was slightly lower than the reference, but without statistical significance (*p* ≥ 0.83). The SBP values recorded by the PulsePen device were slightly higher; however, they were not significant (*p* ≥ 0.99). Nonetheless, the noninvasive central SBP measurement recorded by the Mobil-O-Graph was significantly lower than the reference (mean ± SEM: −7.3 ± 2.7 mmHg, *p* = 0.03).

### 3.4. Correlations between Each Noninvasive and Invasive Central SBP Measurements

The Spearman correlation analysis was performed to verify the association between each noninvasive central SBP and invasive aortic SBP. As shown in Table 3, all noninvasive central SBP measurements, independent of the device or calibration method used, were significantly correlated with the invasive central SBP measurement (*p* < 0.001). The Z-test with Bonferroni adjustment was performed to compare these correlation coefficients. Since comparisons were performed 6 times, a *p*-value less than 0.0083 (0.05/6 ≈ 0.0083) was considered statistically significant. None of the correlation coefficients was significantly different from the others, with a marginal *p*-value in the comparison between the SphygmoCor device using SBP/DBP method and MBP/DBP method (*p* = 0.01).

### 3.5. Pooled Analysis of the Three Potentially Influencing Factors

**Figure 4:**
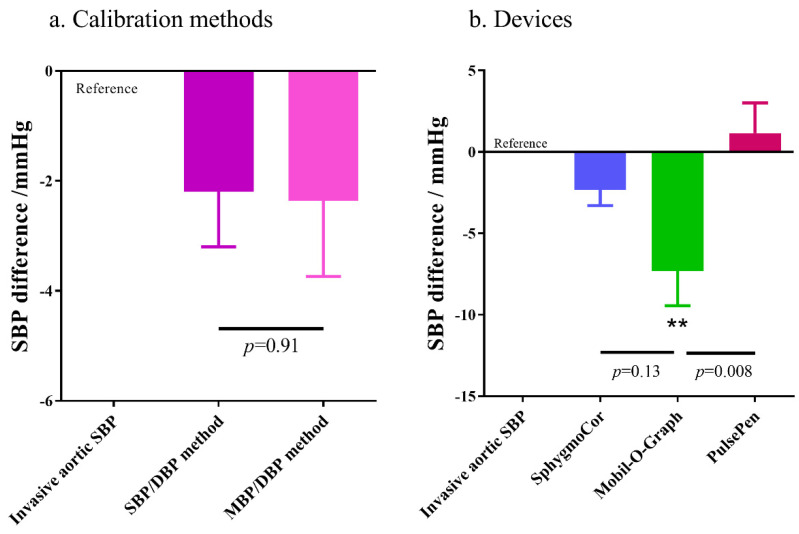
Pooled analysis of the potential influencing factors. All measurements were grouped based on the factors that may potentially impact the accuracy of BP measurement. The difference between each noninvasive measurement and the invasive measurement was calculated and compared. Inva-sive measurement was regarded as the reference “zero”. SBP: systolic blood pressure. DBP: dias-tolic blood pressure. MBP: mean blood pressure. **: *p* < 0.01 compared to the reference.

All measurements were grouped based on the factors that may impact the accuracy of BP measurement, namely calibration methods and devices. The difference between each noninvasive measurement and the invasive measurement was calculated and compared. Figure 4 shows the influence of the two factors on noninvasive BP measurement. Calibration using either the SBP/DBP method or MBP/DBP method was not significantly different when compared to the invasive measurements (*p* ≥ 0.24), and results using these two calibration methods were also similar (*p* = 0.91). As for the devices, results obtained using the SphygmoCor and PulsePen were similar to the invasive measurements (*p* = 0.25 and *p* = 0.59, respectively); meanwhile, results obtained using the Mobil-O-Graph were significantly lower when compared to the invasive measurements (*p* = 0.002) and to the results obtained using the PulsePen (*p* = 0.008), but did not significantly differ from results obtained using the SphygmoCor device (*p* = 0.13).

### 3.6. Multiple Regression Analyses

To further identify independent influencing factors on the accuracy of noninvasive central BP measurement, multiple linear and logistic regression analyses were performed with adjustments for age and gender (Table 4). In multiple linear regression, the difference between invasive and noninvasive measurements was recorded as the dependent variable. The results showed that gender significantly influenced the measurement accuracy (*p* < 0.001). In other words, the noninvasive measurement in women was lower than in men (−0.5 ± 12.2 vs. −3.6 ± 16.2 mmHg, *p* = 0.050). In multiple logistic regression, an absolute difference lower than 5 mmHg was considered an accurate measurement. Similar to linear regression, gender significantly influenced the accuracy of noninvasive central BP measurements (both *p* < 0.001). The calibration method or device did not significantly associate with the accuracy.

## 4. Discussion

Our study demonstrated that different calibration methods, namely the SBP/DBP or MBP/DBP method, were not significantly different in central SBP estimation. Applanation-tonometry-based devices, including the SphygmoCor and the PulsePen, showed good accuracy in BP measurement in our study.

Currently, various noninvasive central BP measurement devices are commercially available. These devices estimate the central BP of different peripheral arteries (for example, brachial or carotid) using different techniques (such as applanation tonometry or oscillometry). There is evidence in the literature that techniques using central BP measurement are a major contributor to the variation noted in the noninvasive measurement of central BP [8]. An important concern in central BP estimation is the appropriate selection of the calibration method in order to obtain the most accurate central BP measurement. Several calibration methods were developed in addition to the transfer function method, for example, the pressure equivalence method [9], the late systolic inflection method [10], and the N-point moving average method [11], among others. Our study focused on the transfer function calibration method (SBP/DBP) and the pressure equivalence calibration method (MBP/DBP). The theoretical basis of the pressure equivalence method is that although SBP varied from the aortic artery to the peripheral artery, MBP and DBP values are consistent in the artery tree [12]. The MBP in this method cannot be simply calculated as 0.33 (or 0.40) * PP + DBP, since this calculation is not able to represent various conditions of waveforms [13]. Although the MBP/DBP method has good accuracy in central BP measurement, it is greatly affected by the SBP amplification [14,15]. Due to the fact that the transfer function method was more convenient and had more validation in different situations than the pressure equivalence method, Kelly et al., who first developed this method, and O’Rourke, the director of the manufacture of the SphygmoCor device, preferred the transfer function method and considered the pressure equivalence method as a good alternative to the transfer function method [16]. Our results confirmed that the pressure equivalence method is as accurate as the transfer function method. However, both methods may underestimate the true aortic BP.

The selection of devices in central BP measurement is important as well. The SphygmoCor device using the generalized transfer function method is the most famous device in central BP measurement. Apart from it, carotid tonometry is another commonly used technique in noninvasive central BP measurement. Carotid BP is a good surrogate to aortic BP because it is anatomically proximal to the aorta. This method was applied in numerous studies, including the Framingham study [17]. However, reproducibility and user experience are barriers to the application of this method in daily practice. Moreover, O’Rourke et al. proposed that there was a systematic error in the carotid tonometry method due to the “Popeye” phenomenon, that is, low amplification of pulse between brachial and aortic arteries and high amplification between radial and aortic arteries (similar to Popeye, whose forearms are extremely hypertrophied) [16]. Our study confirmed the accuracy of the carotid tonometry method using the PulsePen device, and this result was consistent with both calibrated noninvasive brachial BP measurement and calibrated invasive radial BP measurement. Additionally, the carotid tonometry method was the only method that did not tend to underestimate the central BP in our study. More studies, especially in populations with complex situations, are warranted to further support the use of carotid tonometry. Another approach, based on brachial cuff BP measurement, was developed and then validated by Weber et al. in 2011 with the Mobil-O-Graph device [18]. Briefly, it measures brachial BP and waveforms, then estimates central BP with a transfer function-like method. This device has drawn much attention in recent years because it is operator-independent, and it is able to obtain 24 h central BP and record subsequent 24 h central BP variability. However, in our study, the Mobil-O-Graph device significantly underestimated central BP compared to invasive measurements. Several factors may contribute to this issue. First, as described in the results section of this text, the noninvasive brachial BP values measured by the Mobil-O-Graph device were significantly lower than the brachial BP values measured by the Omron device, and the absolute difference was significant (126 ± 19 vs. 140 ± 19 mmHg, *p* < 0.001). Given the fact that brachial BP may explain 90 percent of central BP [19], low estimates of central BP are expected based on this faulty noninvasive brachial BP measurement. Second, in the absence of bone or ligament for flattening the brachial artery, waveforms recorded by brachial tonometry might not be accurate [20]. Third, the software of the Mobil-O-Graph device used in our study only provided the SBP/DBP calibration method. According to recommendations from the ARTERY society, the MBP/DBP calibration method is preferred in noninvasive central BP estimation [7], due to the fact that the measurement obtained using the MBP/DBP method is higher than the measurements obtained using the SBP/DBP method, and the latter method may underestimate true central BP. Thus, the Mobil-O-Graph device may provide a more accurate central BP estimation using the MBP/DBP calibration method, which is available in the newest Mobil-O-Graph software.

Another commonly discussed issue in central BP measurement is the site of peripheral BP measurement. The most commonly used method in central BP measurement is the generalized transfer function method. In this method, central BP is estimated in two parts, correctly measured waveforms and transfer function models. Normally, waveforms are recorded at the radial site, and the calibrated noninvasive peripheral BP measurement is obtained at the brachial site (e.g., SphygmoCor device). The question of whether the peripheral BP measured in daily practice (brachial BP) is well matched to the peripheral BP needed in the transfer function (radial BP) is currently an ongoing concern for researchers in the field. In the 1950s and 1960s, Kroeker et al. [21] and Rowell et al. [22] showed that the brachial BP value was similar to the radial BP value. This finding was confirmed by Kelly et al. with the application of nitroglycerin in 1990 [23]. These results constitute the basis for the use of brachial BP in noninvasive central BP estimation with radial tonometry. However, in recent years, some authors have argued that amplification from the brachial to the radial artery was non-negligible [24]; thus, the use of calibrated brachial BP may cause inaccuracy in noninvasive central BP measurement [25]. Because we cannot obtain the exact transfer function in each device, we did not add this factor to our analysis. However, in our study, noninvasive central BP values using calibrated brachial BP were significantly lower than the invasively measured values, which indicated that current noninvasive central BP measurement with brachial BP instead of radial BP in calibration may underestimate true central BP values.

We also found that, in our study, the absolute difference between invasive central SBP and noninvasive central SBP was significantly lower in men than in women. A study that included over 1800 participants showed that the pulse amplification was significantly higher in men than in women (20 ± 14% vs. 13 ± 12%, *p* < 0.001). Additionally, pulse amplification significantly decreased with age [24]. We believe this difference in amplification must have been taken into consideration in functions. However, the exact amount of this amplification might be different in various populations, and this variance may contribute to the underestimation of central BP in women in our cohort.

There are several limitations to our study. First, the volume of participants is low. The small sample size might not be representative of the entire population and cause potential variability or bias. Second, since we did not have a small enough micromanometer tip catheter or wire, invasive brachial BP measurement could not be accurately obtained in our study. Thus, we could not compare the invasive and noninvasive brachial BP measurements and, subsequently, assess the influence of the noninvasive brachial BP measurement accuracy on central BP estimation. Third, compared to the Omron device, the Mobil-O-Graph had significantly lower brachial SBP records and higher DBP records. This difference may contribute to the difference in central BP measurement. Since we cannot obtain the invasive brachial BP, we did not know which one is more accurate for noninvasive brachial BP measurement.

## 5. Conclusions

Our study demonstrated that noninvasive central BP measurement tended to be slightly lower than invasive BP, though without statistical significance. SphygmoCor and PulsePen devices allow for good accuracy in central BP measurement. Additionally, calibration methods, specifically SBP/DBP and MBP/DBP methods, are acceptable.

## Figures and Tables

**Table 1:** Characteristics of participants.

General Characteristics	
Age (years)	62.4 ± 8.9
Male (n, %)	23 (46)
BMI (kg/m^2^)	25.1 ± 3.0
Current smoker (n, %)	11 (22)
Alcohol (n, %)	7 (14)
Hypertension (n, %)	31 (62)
Anti-hypertensive treatment (n, %)	28 (56)
ACE inhibitor/ARB	15 (30)
Beta-receptor blocker	5 (10)
Calcium channel blocker	15 (30)
Diuretics	5 (10)
Other agents	1 (2)
Diabetes (n, %)	4 (8)
Oral hypoglycemic agents	3 (6)
Insulin	1 (2)
eGFR (mL/min/1.73 m^2^)	117 ± 29

BMI: body mass index. ACE: angiotensin-converting enzyme. ARB: angiotensin receptor blocker. eGFR: estimated glomerular filtration rate.

**Table 2:** Invasive and noninvasive blood pressure parameters.

	Aortic BP		Carotid BP		Peripheral BP *	
	SBP	DBP	SBP	DBP	SBP	DBP
Invasive	127 ± 19	95 ± 14	124 ± 20	91 ± 14	139 ± 26	76 ± 12
SphygmoCor	125 ± 18	77 ± 10	126 ± 21	77 ± 10	--	--
PulsePen	--	--	128 ± 20	76 ± 10	--	--
Mobil-O-Graph	120 ± 16	85 ± 11	--	--	126 ± 19	85 ± 11
Omron	--	--	--	--	140 ± 19	77 ± 10

*: Invasive peripheral BP was radial BP measured in the cath lab. The Omron device was used to record noninvasive brachial BP. Noninvasive aortic and carotid blood pressure shown in this table were measured in conventional methods. For SphygmoCor and PulsePen devices, noninvasive brachial blood pressure measured by the Omron device was used for calibration. The Mobil-O-Graph device calculated aortic blood pressure based on the noninvasive brachial blood pressure recorded by itself. *: Peripheral BP was recorded at the radial artery in invasive measurement, and at the brachial artery in noninvasive measurement. BP: blood pressure. SBP: systolic BP. DBP: diastolic BP.

**Table 3:** Correlations between noninvasive and invasive central SBP and comparison among correlation coefficients.

		SphygmoCor		Mobil-O-Graph	PulsePen
**Correlations**		SBP/DBP	MBP/DBP	SBP/DBP	SBP/DBP
Invasive aortic SBP	R *p*	0.72 <0.001	0.63 <0.001	0.66 <0.001	0.70 <0.001
**Comparison of Correlation Coefficients (Z-Value, *p*-Value)**					
SphygmoCor	SBP/DBP	--			
	MBP/DBP	2.49, 0.01	--		
Mobil-O-Graph	SBP/DBP	0.81, 0.42	−0.40, 0.69	--	
PulsePen	SBP/DBP	0.31, 0.76	−1.00, 0.32	−0.47, 0.64	--

Spearman correlation analyses were performed to test the association between each noninvasive central SBP and invasive aortic SBP, and Z-test was used to compare these correlation coefficients. Since comparisons were performed 6 times, a *p*-value less than 0.05/6 ≈ 0.008 was considered sta-tistically significant. Thus, none of these Z-tests achieved statistically significance. SBP: systolic blood pressure. DBP: diastolic blood pressure.

**Table 4:** Multiple regression analyses between measurement accuracy and potential impacting factors.

Multiple Linear Regression (Independent Variable: Difference between Invasive and Noninvasive Measurements)			
	β	SE	*p*
age (+ 1 year)	−0.07	0.06	0.27
gender (male = 1, female = 0)	−4.22	1.11	<0.001
calibration method (SBP/DBP = 1, MBP/DBP = 0)	−0.48	1.33	0.72
device (SphygmoCor = 1, others = 0)	−2.17	1.26	0.09
Multiple logistic regression (independent variable: accuracy (difference < 5 mmHg) of noninvasive measurement)			
	OR	95% CI	*p*
age (+ 1 year)	1.00	0.97, 1.03	0.99
gender (male = 1, female = 0)	0.41	0.25, 0.69	<0.001
calibration method (SBP/DBP = 1, MBP/DBP = 0)	0.63	0.33, 1.23	0.18
device (SphygmoCor = 1, others = 0)	1.14	0.65, 2.02	0.64

The difference (absolute value) between invasive and noninvasive measurements was calculated. If the difference is less than 5 mmHg, this noninvasive measurement was regarded as an accurate measurement. Full-model multiple regression analyses were performed to assess the relationship between the absolute difference (linear regression)/accuracy (logistic regression) and potential confounders including age, gender, calibration method, and device. Gender, calibration method, and device were regarded as categorical variables. Dummy variables were introduced, and men, SBP/DBP calibration, and SphygmoCor were taken as the references, respectively. SE: standard error. OR: odds ratio. CI: confidence interval. SBP: systolic blood pressure. DBP: diastolic blood pressure.

## Data Availability

The data presented in this study are available on request from the corresponding author. The data are not publicly available due to privacy.

## References

[1] Williams B., Mancia G., Spiering W., Agabiti Rosei E., Azizi M., Burnier M., Clement D.L., Coca A., de Simone G., Dominiczak A. (2018). 2018 ESC/ESH Guidelines for the management of arterial hypertension: The Task Force for the management of arterial hypertension of the European Society of Cardiology and the European Society of Hypertension. J. Hypertens..

[2] Peluso G., Garcia-Espinosa V., Curcio S., Marota M., Castro J., Chiesa P., Giachetto G., Bia D., Zocalo Y. (2017). High Central Aortic Rather than Brachial Blood Pressure is Associated with Carotid Wall Remodeling and Increased Arterial Stiffness in Childhood. High Blood Press. Cardiovasc. Prev. Off. J. Ital. Soc. Hypertens..

[3] Chi C., Yu X., Auckle R., Lu Y., Fan X., Yu S., Xiong J., Bai B., Teliewubai J., Zhou Y. (2017). Hypertensive target organ damage is better associated with central than brachial blood pressure: The Northern Shanghai Study. J. Clin. Hypertens. (Greenwich Conn.).

[4] Booysen H.L., Norton G.R., Maseko M.J., Libhaber C.D., Majane O.H., Sareli P., Woodiwiss A.J. (2013). Aortic, but not brachial blood pressure category enhances the ability to identify target organ changes in normotensives. J. Hypertens..

[5] Hashimoto J. (2017). Central Hemodynamics for Management of Arteriosclerotic Diseases. J. Atheroscler. Thromb..

[6] Cheng H.M., Lang D., Tufanaru C., Pearson A. (2013). Measurement accuracy of non-invasively obtained central blood pressure by applanation tonometry: A systematic review and meta-analysis. Int. J. Cardiol..

[7] Sharman J.E., Avolio A.P., Baulmann J., Benetos A., Blacher J., Blizzard C.L., Boutouyrie P., Chen C.H., Chowienczyk P., Cockcroft J.R. (2017). Validation of non-invasive central blood pressure devices: ARTERY Society task force consensus statement on protocol standardization. Eur. Heart J..

[8] Narayan O., Casan J., Szarski M., Dart A.M., Meredith I.T., Cameron J.D. (2014). Estimation of central aortic blood pressure: A systematic meta-analysis of available techniques. J. Hypertens..

[9] Kelly R., Fitchett D. (1992). Noninvasive determination of aortic input impedance and external left ventricular power output: A validation and repeatability study of a new technique. J. Am. Coll. Cardiol..

[10] Pauca A.L., O’Rourke M.F., Kon N.D. (2001). Prospective evaluation of a method for estimating ascending aortic pressure from the radial artery pressure waveform. Hypertension.

[11] Williams B., Lacy P.S., Yan P., Hwee C.N., Liang C., Ting C.M. (2011). Development and validation of a novel method to derive central aortic systolic pressure from the radial pressure waveform using an n-point moving average method. J. Am. Coll. Cardiol..

[12] Pauca A.L., Wallenhaupt S.L., Kon N.D., Tucker W.Y. (1992). Does radial artery pressure accurately reflect aortic pressure?. Chest.

[13] O’Rourke M.F., Adji A. (2010). Clinical use of applanation tonometry: Hope remains in Pandora’s box. J. Hypertens..

[14] Picone D.S., Schultz M.G., Peng X., Black J.A., Dwyer N., Thomson P.R., Qasem A., Sharman J.E. (2019). Intra-arterial analysis of the best calibration methods to estimate aortic blood pressure. J. Hypertens..

[15] Schultz M.G., Picone D.S., Armstrong M.K., Black J.A., Dwyer N., Thomson P.R., Sturgess D., Sharman J.E. (2020). The influence of SBP amplification on the accuracy of form-factor-derived mean arterial pressure. J. Hypertens..

[16] O’Rourke M.F., Adji A. (2012). Noninvasive studies of central aortic pressure. Curr. Hypertens. Rep..

[17] Mitchell G.F., Hwang S.J., Vasan R.S., Larson M.G., Pencina M.J., Hamburg N.M., Vita J.A., Levy D., Benjamin E.J. (2010). Arterial stiffness and cardiovascular events: The Framingham Heart Study. Circulation.

[18] Weber T., Wassertheurer S., Rammer M., Maurer E., Hametner B., Mayer C.C., Kropf J., Eber B. (2011). Validation of a brachial cuff-based method for estimating central systolic blood pressure. Hypertension.

[19] Cameron J.D., McGrath B.P., Dart A.M. (1998). Use of radial artery applanation tonometry and a generalized transfer function to determine aortic pressure augmentation in subjects with treated hypertension. J. Am. Coll. Cardiol..

[20] O’Rourke M.F., Safar M.E., Roman M.J. (2010). Letter by O’Rourke et al regarding article, “Arterial stiffness and cardiovascular events: The Framingham Heart Study”. Circulation.

[21] Kroeker E.J., Wood E.H. (1955). Comparison of simultaneously recorded central and peripheral arterial pressure pulses during rest, exercise and tilted position in man. Circ. Res..

[22] Rowell L.B., Brengelmann G.L., Blackmon J.R., Bruce R.A., Murray J.A. (1968). Disparities between aortic and peripheral pulse pressures induced by upright exercise and vasomotor changes in man. Circulation.

[23] Kelly R.P., Gibbs H.H., O’Rourke M.F., Daley J.E., Mang K., Morgan J.J., Avolio A.P. (1990). Nitroglycerin has more favourable effects on left ventricular afterload than apparent from measurement of pressure in a peripheral artery. Eur. Heart J..

[24] Segers P., Mahieu D., Kips J., Rietzschel E., De Buyzere M., De Bacquer D., Bekaert S., De Backer G., Gillebert T., Verdonck P. (2009). Amplification of the pressure pulse in the upper limb in healthy, middle-aged men and women. Hypertension.

[25] Davies J.E., Shanmuganathan M., Francis D.P., Mayet J., Hackett D.R., Hughes A.D. (2010). Caution using brachial systolic pressure to calibrate radial tonometric pressure waveforms: Lessons from invasive study. Hypertension.

